# An Index of Materia Medica

**Published:** 1888-02

**Authors:** 


**An Index of Materia Medica.** By CHAS. H. MAY, M. D., Instructor in Ophthalmology, New York Polyclinic, and CHAS. F. MASON, M. D., Assistant Surgeon, U. S. A. Wm. Wood & Co.

This little book belongs to Wood's series of pocket manuals. It contains a list of the pharmacopœal preparations, with doses, solubility, etc., together with some practical hints in prescription-writing. The book is interleaved and well adapted to serve as a note-book for students of materia medica. It is to be regretted that the authors have made the attempt to give instructions in orthoepy as well as materia medica, for they have managed to misplace their accents quite as frequently as they have succeeded in placing them in their proper positions. The subject of the pronunciation of medical terms is already in a sufficiently chaotic state, without placing such books as this in the hands of students to render their confusion worse confounded.

